# Genome-Wide Transcriptional Analysis and Functional Validation Linked a Cluster of Epsilon Glutathione S-Transferases with Insecticide Resistance in the Major Malaria Vector *Anopheles funestus* across Africa

**DOI:** 10.3390/genes12040561

**Published:** 2021-04-13

**Authors:** Mersimine F. M. Kouamo, Sulaiman S. Ibrahim, Jack Hearn, Jacob M. Riveron, Michael Kusimo, Magellan Tchouakui, Terence Ebai, Williams Tchapga, Murielle J. Wondji, Helen Irving, Thaddée Boudjeko, Fabrice F. Boyom, Charles S. Wondji

**Affiliations:** 1 LSTM Research Unit, Centre for Research in Infectious Diseases (CRID), Yaoundé P.O. Box 13591, Cameroon; sulaimansadi.ibrahim@crid-cam.net (S.S.I.); gkusimo@gmail.com (M.K.); magellan.tchouakui@crid-cam.net (M.T.); ebai.terence@crid-cam.net (T.E.); williams.tchapga@crid-cam.net (W.T.); murielle.wondji@lstmed.ac.uk (M.J.W.); 2 Laboratory of Phytoprotection and Plant Valorisation (LPVRV), Biotechnology Centre, University of Yaoundé 1, Yaoundé P.O. Box 812, Cameroon; boudjeko@yahoo.com; 3 Department of Biochemistry, Faculty of Science, University of Yaoundé 1, Yaoundé P.O. Box 812, Cameroon; fabrice.boyom@fulbrightmail.org; 4 Antimicrobial and Biocontrol Agents Unit, Laboratory for Phytobiochemistry and Medicinal Plants Studies, University of Yaoundé I, Yaoundé P.O. Box 812, Cameroon; 5 Department of Biochemistry, Bayero University, Kano PMB 3011, Nigeria; 6 Department of Vector Biology, Liverpool School of Tropical Medicine, Pembroke Place, Liverpool L35QA, UK; jack.hearn@lstmed.ac.uk (J.H.); jacob.riveron_miranda@syngenta.com (J.M.R.); helen.irving@lstmed.ac.uk (H.I.)

**Keywords:** malaria, *Anopheles funestus*, metabolic resistance, glutathioneS-transferase, RNA interference

## Abstract

Resistance is threatening the effectiveness of insecticide-based interventions in use for malaria control. Pinpointing genes associated with resistance is crucial for evidence-based resistance management targeting the major malaria vectors. Here, a combination of RNA-seq based genome-wide transcriptional analysis and RNA-silencing in vivo functional validation were used to identify key insecticide resistance genes associated with DDT and DDT/permethrin cross-resistance across Africa. A cluster of glutathione-S-transferase from epsilon group were found to be overexpressed in resistant populations of *Anopheles funestus* across Africa including *GSTe1* [Cameroon (fold change, FC: 2.54), Ghana (4.20), Malawi (2.51)], *GSTe2* [Cameroon (4.47), Ghana (7.52), Malawi (2.13)], *GSTe3* [Cameroon (2.49), Uganda (2.60)], *GSTe4* in Ghana (3.47), *GSTe5* [Ghana (2.94), Malawi (2.26)], *GSTe6* [Cameroun (3.0), Ghana (3.11), Malawi (3.07), Uganda (3.78)] and *GSTe7* (2.39) in Ghana. Validation of *GSTe* genes expression profiles by qPCR confirmed that the genes are differentially expressed across Africa with a greater overexpression in DDT-resistant mosquitoes. RNAi-based knock-down analyses supported that five *GSTe* genes are playing a major role in resistance to pyrethroids (permethrin and deltamethrin) and DDT in *An. funestus*, with a significant recovery of susceptibility observed when *GSTe2*, *3*, *4*, *5* and *GSTe6* were silenced. These findings established that *GSTe3*, *4*, *5* and *6* contribute to DDT resistance and should be further characterized to identify their specific genetic variants, to help design DNA-based diagnostic assays, as previously done for the *119F-GSTe2* mutation. This study highlights the role of *GSTe*s in the development of resistance to insecticides in malaria vectors and calls for actions to mitigate this resistance.

## 1. Introduction

Malaria is the deadliest vector-borne disease, killing more than 400,000 people every year [1]. Vector control interventions through the use of long-lasting insecticide nets and the implementation of indoor residual spray have led to a significant reduction in malaria incidence, between 2000 and 2015 [2]. This gain is under threat, as the most recent WHO World Malaria Report revealed there has been increase in annual case numbers since 2016. This malaria rebound is partly due to escalation of insecticide resistance in major malaria vectors such as *An. gambiae* and *An. funestus*. This was recently shown for a population of *An. funestus* for which high resistance level to pyrethroids was associated with a significant loss of efficacy of insecticide-treated nets including PBO-based nets [3]. The main mechanisms of resistance are target-site and metabolic resistance. The molecular basis of metabolic resistance is more complex and involves among others an overexpression and/or over-activity of major detoxification genes, such as cytochrome P450s (CYP450s), glutathione-S-transferases (GSTs) and the carboxylesterases [4,5,6], in addition to the recently described sensory appendages proteins [7]. Several studies, including genome-wide transcriptional analyses using microarray/qPCR and functional validation have linked GST with resistance in the major malaria vectors [8,9,10]. However, most of these studies have concentrated on *GSTe2*, neglecting the other *GST epsilon* genes, even though they have been shown to also consistently be overexpressed [11,12,13,14,15]. Indeed, among the 8 potential members of the GST epsilon class, several genes have previously been shown to be overexpressed in malaria vectors including *An. gambiae* [10] and *An. funestus* [14,15,16]. However, apart from few validations such as for *GSTe4* in *An. gambiae* and *An. arabiensis* [17] little is known on the role of these genes in insecticide resistance. In the case of *An. funestus*, progress made has focused on the *GSTe2* gene detecting a key resistance marker (*L119F-GSTe2*) now commonly used for resistance monitoring in *An. funestus* [16]. In addition to conferring pyrethroid/DDT resistance, it was also shown that the *L119F-GSTe2*-mediated metabolic resistance to pyrethroids/DDT is associated with negative effects on some life-history traits of field populations of *An. funestus*, supporting that insecticide resistance is associated with a fitness cost [18]. An experimental hut study, using the same marker in *An. funestus* population from Mibellon (Cameroon) had confirmed that presence of the *L119F-GSTe2* was associated with resistance to DDT and pyrethroids [19] and was reducing the efficacy of bed nets [20].

RNA interference (RNAi) is one of the main approaches commonly used for *in vivo* validation of the role of detoxification enzymes in conferring resistance to insecticides in mosquitoes. This is done by injecting adult mosquitoes with double-stranded RNA (dsRNA) corresponding to the gene of interest. In turn, this induces mRNA degradation via the RNAi pathway and suppresses expression of the protein [21,22]. RNAi has been used to link overexpression of cytochrome P450s and GSTs to insecticide resistance in *An. gambiae* and *Aedes* mosquitoes [23,24,25]. Recently, RNAi has been used to establish the role of sensory appendage proteins in the leg of *An. gambiae* in conferring pyrethroid resistance [7] confirming that this method provides a robust approach to validate the contribution of specific genes such as those of the GST epsilon, to a specific phenotype.

In this study, using a genome-wide RNA-seq-based transcriptomic analysis, we detected candidate genes associated with DDT-resistance and DDT/permethrin cross-resistance across different regions of Africa revealing the pre-eminence of GST epsilon genes. The role of epsilon class of GSTs in DDT/pyrethroid resistance was investigated in *An. funestus* population from Mibellon (Cameroon) using RNAi-mediated gene silencing. The results suggest that several members of this class in addition to *GSTe2* contribute to the overall resistance observed in the field.

## 2. Materials and Methods

### 2.1. Mosquito Collection and Rearing

Indoor-resting female *An. funestus* were collected early in the morning (6:00 a.m.–8:00 a.m.), using battery-powered aspirators (John. W. Hock, Gainesville, FL, USA) in Mibellon (6°46′ N, 11°70′ E, Cameroon; 2015 and 2019), Obuasi (5°56′ N, 1°37′ W, Ghana; 2014), Kpome (6°55′ N, 2°19′ E, Benin, 2014), Chikwawa (16°1′ S, 34°47′ E, Malawi; 2014), and Tororo (0°45′ N, 34°5′ E, Uganda; 2014). The collection was done from randomly selected houses, following a verbal consent from the chief of the district and the household owners. Mosquitoes collected were kept in paper cups, being transported to the insectary in the Centre for Research in Infectious Diseases (CRID), Yaoundé, Cameroon. The F_1_ generation was generated in the insectary from field blood-fed female mosquitoes using forced-egg laying method [26]. Briefly, F_0_ gravid females were transferred into 1.5 mL Eppendorf tubes containing a wet filter paper, to enable them to lay eggs. After oviposition the parents that laid eggs were removed and used for species identification. Molecular identification to species level was carried out according to the protocol described priviously [27] using genomic DNA (gDNA) extracted from F_0_ females identified morphologically as *An. funestus*. DNA extraction was done using the Livak protocol [28]. The eggs were pooled into bowls and supplemented with TetraMin™ baby fish food. The emerged F_1_ female progenies were mixed in cages and 2 to 5-day old females used for insecticide bioassays and double-stranded RNA (dsRNA) injection.

### 2.2. Transcriptomic Profiling of DDT Resistance across Africa Using RNA-Seq

Transcriptional profiling of *An. funestus* populations was carried out to detect key candidate genes associated with DDT resistance across Africa. This was done using mosquitoes from four African regions: Central (Mibellon/Cameroon), West (Obuasi/Ghana), Southern (Chikwawa/Malawi), and East (Tororo/Uganda). Total RNA was extracted from pools of 10 female DDT-resistant mosquitoes (alive after 24 h exposure to DDT), unexposed mosquitoes (control) and lab susceptible colony (FANG) using the Arcturus PicoPure RNA Isolation Kit (Life Technologies, Carlsbad, CA, USA) according to the manufacturer’s instructions. The FANG is a fully insecticide susceptible laboratory colony [29]. In addition, RNA was extracted from 10 permethrin-resistant mosquitoes (alive after 24 h exposure to permethrin) to study the gene differentially expressed across Africa when comparing DDT vs. permethrin-resistant mosquitoes.

RNA libraries were pooled in equimolar amounts using the Qubit and Bioanalyzer data. The quantity and quality of each pool were assessed by Bioanalyzer and subsequently by qPCR using the Kapa Illumina library quantification kit (Kapa Biosystems, Wilmington, MA, USA), on a Light Cycler LC480II (Roche, Basel, Switzerland), according to manufacturers’ instructions. The pool of libraries was sequenced on one lane of the HiSeq 2500 (Illumina, San Diego, CA, USA) at 2 × 125 bp paired-end sequencing with v4 chemistry. Sequence library preparation, sequencing, initial processing and quality control were done by the Centre for Genomic Research, University of Liverpool, UK. Alignment to the reference sequence using the AfunF3.1 annotation. (https://vectorbase.org/vectorbase/app/record/dataset/DS_1a787d4361#pmids, accessed on 7 April 2020). Data were analysed as described previously [30,31,32]. Differential gene expression analysis was performed using edgeR and the Strand NGS program (Strand Life Sciences, version 3.0, Hebbal, Bangalore, India).

### 2.3. Investigation of Expression Profile of GSTe Genes in An. funestus across Africa

The expression profiles of *GSTe1*, *GSTe2*, *GSTe3*, *GSTe4*, *GSTe5*, *GSTe6*, *GSTe7* and *GSTe8* in DDT and permethrin-resistant mosquitoes was assessed across Africa (Cameroon, Benin, Malawi, and Uganda), using qRT-PCR. RNA was extracted from three biological replicates from each population of 10 each of DDT-resistant females (alive 24 h after exposure to DDT), permethrin-resistant (alive 24 h after exposure to permethrin), control (*An. funestus* mosquitoes not exposed to any insecticide), as well as susceptible laboratory colony (FANG). Briefly, 1 μg of the total RNA from each of the three biological replicates was used as the template for cDNA synthesis using Superscript III (Invitrogen, Carlsbad, CA, USA) with oligo-dT20 and RNase H, according to the manufacturer’s instructions. The qRT-PCR amplification was performed as described [15] using the primers provided in Supplementary Table S2.1. The relative expression and fold change of each *GSTe* gene was calculated as previously described [33] by comparing expression in resistant, susceptible and control samples. The normalization was done with the ribosomal protein S7, *RPS7* (AFUN007153) and *actin5C* (AFUN006819) housekeeping genes.

### 2.4. Functional Validation of Role of GSTe Genes in Resistance Using RNA Interference

#### 2.4.1. Double Strand RNA Synthesis

Double-stranded RNAs specific to *GSTe* genes of interest were synthesized for use in RNAi gene-silencing experiments. Each *GSTe* oligonucleotide primer was designed using specific cDNA of the corresponding genes downloaded from the Vector Base (https://vectorbase.org/vectorbase/app, accessed on 8 December 2016). The T7 RNA polymerase promoter sequence, TAATACGACTCACTATAGGGAGA, was added to the 5′ end of each primer (Supplementary Table S2.2). Specific *GST2*, *3*, *4*, *5*, *6*, *7* and *GSTe8* fragments were amplified by PCR from plasmid clones using KAPA Taq Kit (Kapa Biosystems, Wilmington, MA USA). Double-stranded RNA (dsRNA) was synthesized using in vitro transcription MEGAscript^®®^ T7 Kit (Ambion Inc., Austin, TX, USA) and purified using MEGAclear columns (Ambion). The purified products were concentrated by ethanol precipitation and the dsRNA was resuspended in nuclease-free water and stored at −20 °C. The successful construction of dsRNA was confirmed by running 3 μL of dsRNA-diluted products in 1.5% agarose gel in a Tris-acetate-EDTA (TAE) buffer.

#### 2.4.2. Mosquitoes Injection and Susceptibility Bioassays

To explore the role of *GSTe* genes in conferring insecticide resistance, RNAi was performed on Mibellon *An. funestus* population, by injecting sequence-specific dsRNA to 2–3 days old F_1_ female mosquitoes, followed by insecticide bioassay. A Nano injector (Nanoinject; Drummond, Burton, OH, USA) was used to inject *dsGSTe2*, *3*, *4*, *5*, *6*, *7* and *dsGSTe8* into the thorax of 2 to 3 days old female *An. funestus* mosquitoes as described [34]. Briefly, mosquitoes, induced to sleep with CO_2_, were injected with 69 nL of either aliquot of above *dsGSTes* or *dsGFP* (control). Four days after injection, four replicates of 20 mosquitoes for each dsRNA were exposed to permethrin (0.75%), deltamethrin (0.05%) and DDT (4%) for 1 h following the WHO testing protocol [35]. Mosquitoes were transferred to holding tubes after exposure, supplemented with sugar and mortalities counted 24 h after the exposure. The susceptibility test was performed in triplicate with experimental mosquitoes comprising the mosquitoes injected with *dsGSTes* above, whereas mosquitoes injected with *dsGFP* and those not injected were used as controls.

#### 2.4.3. Quantitative RT-PCR to Confirm the Knockdown Effect

For *dsGSTe*-injected and non-injected mosquitoes, RNA was extracted from 3 pools of 5 mosquitoes using TRIzol reagent (Gibco BRL, Gaithersburg, MD, USA). cDNA from each of the three biological replicates was synthesized using the Super-Script III (Invitrogen, Carlsbad, CA, USA) with oligo-dT20 and RNase H, according to the manufacturer’s instructions. The cDNA from each replicate treatment was then used to assess the extent of RNAi by measuring levels of gene expression after injection by qRT-PCR. To assess the knockdown efficiency after injection and quantitative difference in the level of *GSTes* expression between injected and non-injected mosquitoes, a standard curve of each gene was established using a serial dilution of cDNA. The qPCR amplification was carried out in a MX3005 real-time PCR system using Brilliant III Ultra-Fast SYBR Green qPCR Master Mix (Agilent, Santa Clara, CA, USA). A total of 10 ng of cDNA from each sample was used as a template in a three-step program involving a denaturation at 95 °C for 3 min followed by 40 cycles of 10 s at 95 °C and 10 s at 60 °C and a last step of 1 min at 95 °C, 30 s at 55 °C and 30 s at 95 °C. The relative expression and fold-change of each target gene were calculated according to the 2^−ΔΔCT^ Livak method [33], comparing expression in specific *dsGSTe*-injected samples to non-injected ones, after normalization with the housekeeping genes, *RPS7* (AFUN007153) and *actin5C* (AFUN006819), as described above.

### 2.5. Data Analysis

All analyses were conducted using GraphPad Prism version 7.00, R 3.3.2. for Windows and Strand NGS program (Strand Life Sciences, version 3.0, Hebbal, Bangalore, India). Students’ *t*-test was used to assess statistical differences between experimental and control groups.

## 3. Results

### 3.1. RNAseq-Based Comparative Transcriptomic Profiling of DDT Resistance across Africa

**Figure 1:**
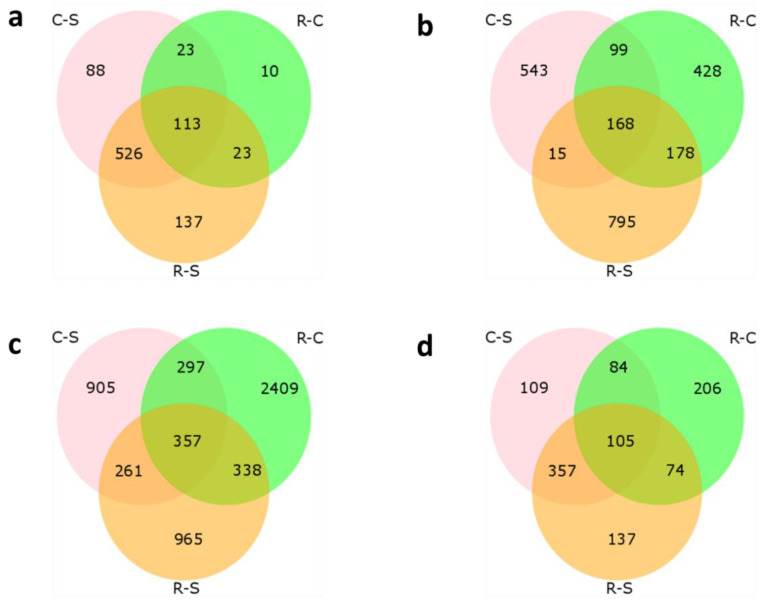
Venn diagrams showing the number of differentially expressed genes in each population compared to the FANG susceptible colony: (**a**) Cameroon population, (**b**) Ghana population, (**c**) Uganda population and (**d**) Malawi population. R-C: genes induced upon exposure and constitutive expression (resistant vs control unexposed), C-S: constitutive differential expression (control vs susceptible); R-S: genes induced upon exposure and constitutive expression (resistant vs susceptible).

To detect genes associated with DDT resistance in *An. funestus* mosquitoes Africa-wide, transcriptional profiling of mosquitoes from different regions of Africa was performed. This comprised populations from southern (Malawi), East (Uganda), West (Ghana) and Central (Cameroon) Africa. Priority was given to the comparison between genes upregulated in DDT-resistant mosquitoes (R) and the control (C, unexposed mosquitoes) because this comparison directly focuses on the difference between mosquitoes having the same genetic background (accounting for potential induction of expression), but differing in treatment received. Attention was also given to genes that were commonly upregulated in R vs Susceptible FANG colony mosquitoes (S) and C vs S as these genes are the ones expressed constitutively in natural mosquitoes’ populations. The number of differentially expressed genes in each of the four populations and the FANG susceptible strain is shown in Venn diagrams (Figure 1). Raw data from RNA-seq is deposited on sequence archive, with the following link: https://www.ebi.ac.uk/ena/browser/view/PRJEB24351, accessed on 10 January 2018.

#### 3.1.1. The Central Africa Population of Cameroon

The major detoxification gene families found to be overexpressed in Cameroon population are cytochrome P450s, glutathione S-transferases and carboxylesterases. When comparing resistant, susceptible and control mosquito cytochrome P450 *CYP325A* is over-expressed, followed by *CYP6P9b*, *CYP6P5*, *CYP315A1*. For GSTs, the epsilon and delta family are upregulated when comparing R-S vs. C-S expression profile. The most overexpressed GSTs are *GSTe2*, *GSTe1*, *GSTe3*, *GSTe6*, *GSTd3* and *GSTt2*. Several genes from carboxylesterase classes, e.g., an unknown COE (AFUN002514), and *COEBE3C* (AFUN016311, a glutathione peroxidase (AFUN022201), an ATP-binding cassette transporter (AFUN019220), a UDP-glucuronosyltransferase (AFUN011266), and sulfotransferase family (AFUN016207) (*SULT1B*), were also found to be overexpressed (Table 1).

#### 3.1.2. The West African Population of Ghana

Many cytochrome P450s were found to be overexpressed when comparing expression profiles of R, C and S in mosquitoes from Ghana. These include *CYP6P4a* (AFUN020895), *CYP325B/C*, *CYP4H26*, *CYP6M4*, *CYP4C36*, *CYP9K1*, *CYP9J5*, *CYP6P9b*, *CYP6P9a*, *CYP6P5* and *CYP4H17* (Table 2). In Ghana, almost all the GST epsilon clusters, *GSTe1*, *GSTe2*, *GSTe3*, *GSTe4*, *GSTe5*, *GSTE6* and *GSTe7,* are upregulated in C and R samples. We also note the overexpression of a sulfotransferase *SULT1B*, D7 short form salivary protein (AFUN016458), UDP-glucuronosyltransferase (AFUN011266), carboxylesterase (AFUN016367) and ATP-binding cassette transporter (AFUN019220).

#### 3.1.3. The Southern Africa Population of Malawi

The most overexpressed genes in the Malawi population when comparing R to S are the two P450s *CYP6P9a* and *b* (Table 3). Besides those two genes, many other P450s, including *CYP325J1*, *CYP6M4*, *CYP6P2*, *CYP9K1*, *CYP314A1*, *CYP6N1* and cytochrome *b5*, are upregulated. For GSTs, the most overexpressed genes are from delta family, e.g., *GDTD1* and *GSTD11*. The GST epsilon family include *GSTe1*, *GSTe2*, *GSTe5* and *GSTe6*. In addition, theta family *GSTt1* was also found to be overexpressed in exposed mosquitoes. As for Central and West Africa, we also have the overexpression of carboxylesterase (AFUN016265), sulfotransferase (AFUN016207) and ATP-binding cassette transporter (AFUN019220).

#### 3.1.4. The East African Population of Uganda

Here, the most overexpressed genes are cytochrome P450s, with *CYP4C26*, *CYP6P5*, *CYP6P4a*, *CYP306A1*, *CYP305A3* and *CYP315A1*. Some GST families are also significantly over-expressed when comparing R to C mosquitoes. These include *GSTD1*, *GSTD3* and *GSTe6*. However, not many GST epsilon genes are overexpressed in Uganda compared to other Africa countries, e.g., Cameroon and Ghana. Many other genes families, such as carboxyesterases, sulfotransferase, NADHP, and ATP-binding cassette transporter, are expressed in Uganda (Table 4).

Noticeably, the analysis of gene expression across Africa between DDT-resistant mosquito population revealed that GSTs are upregulated across the continent. The level of expression of GSTs is variable across the continent and three major families, the epsilon, the delta and the theta family, are the most overexpressed. Regarding the GST epsilon cluster specifically, we observed that all genes are up-regulated across Africa when comparing control mosquitoes versus DDT-resistant population, except for *GSTe8*.

#### 3.1.5. Genes Differentially Expressed across Africa when Comparing DDT vs. Permethrin-Resistant Mosquitoes

- Cameroon (Central Africa):

Analysis of the genes significantly upregulated in DDT-resistant mosquitoes when comparing to permethrin-resistant mosquitoes revealed the presence of CYP325A (FC: 1.56), some eukaryotic large and small subunit ribosomal RNA (AFUN017765) (FC: 24), secretory phospholipase A2 (AFUN022209) (FC: 1.7), and D7 short form salivary protein (AFUN016457) (FC: 1.6). Some genes including the probable prefoldin subunit PFD6 (FC: −2.07), odorant-binding protein (AFUN002277) (FC: −2.6) and Hexamerin (AFUN018859) (FC: −2.51) are rather significantly downregulated in permethrin-resistant mosquitoes (Supplementary Table S1.1).

- Ghana (West Africa):

Analysis has shown that no upregulated detoxification enzyme family such as cytochrome P450s, glutathione-S-transferase or carboxylesterase is significantly over-expressed in DDT-resistant mosquitoes when compared to permethrin-resistant. However, some other genes with no known detoxification role including haemolymph protein (AFUN021325) *P27K* (FC 1.5), heat shock protein (AFUN019847) *HSP90* (FC: 1.62), and transcription initiation factor (AFUN019720) *TAF13* (FC 1.56) are upregulated in DDT-resistant mosquitoes. However, *CYP4H19* is downregulated in permethrin-resistant mosquitoes (FC: −1.54) (Supplementary Table S1.2).

- Malawi (Southern Africa):

When comparing DDT vs. permethrin-resistant populations in Malawi some detoxification genes including *CYP6P9b* (FC: 1.99), *GSTU3* (FC: 1.67), glutathione peroxidase (AFUN022201) (FC: 1.8), as well as serine proteinase (AFUN022250) (FC: 5.9) and the probable prefoldin subunit 6 (AFUN021561) (FC: 3.4) are overexpressed in DDT-resistant mosquitoes. Other genes including carboxylic ester hydrolase *COEBE2C* (AFUN016052) (FC: −3.21), *CYP4H25* (FC: −1.74), *GSTD11* (FC: −1.80) and *GSTU2* (FC: −1.64) are downregulated in permethrin-resistant mosquitoes (Supplementary Table S1.3).

- Uganda (East Africa):

Comparison shows that some genes such as carboxylesterase (AFUN016265) (FC 1.77), Putative serine protease (AFUN016153) (FC 2.87), and the eukaryotic large and small subunit ribosomal RNA (AFUN017404, AFUN017328,) (with, respectively, fold change 47.04 and 3.20) are upregulated in DDT-resistant mosquitoes. Some detoxification genes including cytochromes P450 (*CYP325J1*, *CYP325Z1*, *CYP6Z4*), Zinc metalloproteinase nas-1(AFUN021369) (FC 2.91), UDP-glucuronosyltransferase (AFUN000679) (FC: −1.75), Lactosylceramide 4-α-galactosyltransferase (AFUN020965) (FC: −3.27), and Heat shock protein 70 B2 (AFUN019513) (FC: −4.58) (Supplementary Table S1.4) are rather downregulated in permethrin-resistant mosquitoes.

### 3.2. qPCR Transcriptional Profiling of GSTe Genes in An. funestus across Africa

**Figure 2:**
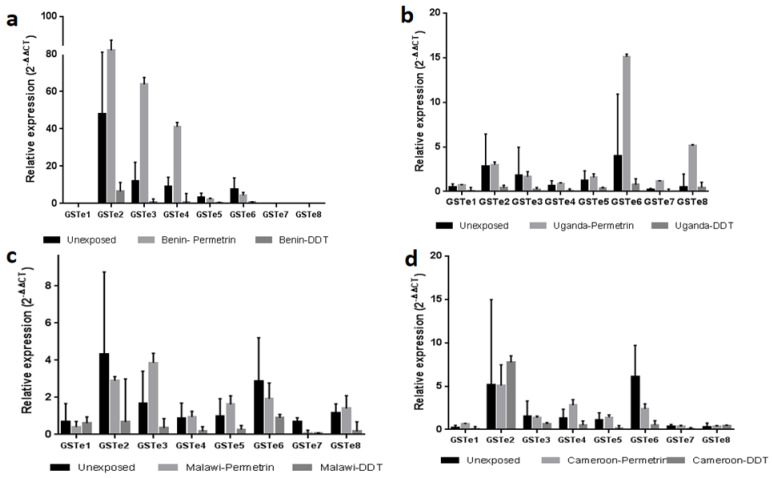
Evaluation of *GSTe* expression profile across Africa by qPCR. (**a**) *GSTe* expression profiles in Benin: *GSTe2*, *3* and *4* are the more expressed (**b**) *GSTe*s expression profile in Uganda: *GSTe6*, *8*, *2* and *3* are the more expressed; (**c**) *GSTe*s expression profile in Malawi: *GSTe2*, *3*, *6*, *5* and *8* are the more expressed; (**d**) *GSTe*s expression profile in Cameroon: *GSTe2 6*, *4*, *3* and *GSTe5* are the more expressed. Consistent with RNA-seq data, GST epsilons are differentially expressed, and expression level is higher in DDT-resistant mosquitoes than the control in general.

The validation of the expression profile of genes of the *GSTe* cluster across the continent was carried out using qPCR, comparing the expression level of *GSTe*s between FANG, unexposed (control), permethrin-alive and DDT-alive mosquitoes after 24 h exposure. In Benin, the level of expression of *GSTe2*, *GSTe3* and *GSTe4* was significantly higher in mosquitoes resistant to DDT compared to the unexposed group and the permethrin-resistant mosquitoes (Figure 2). In Uganda, all the *GSTe*s were expressed but at comparatively the same level, with the most over-expressed one being *GSTe6*, followed by *GSTe*8. As observed with Benin samples, these two genes were more expressed in mosquitoes surviving DDT exposure. In Malawi, it was observed in ascending order that the most expressed *GSTe*s were *GSTe2*, *GSTe3*, *GSTe6*, *GSTe5* and *GSTe8*. However, except for the *GSTe6*, which was more overexpressed in mosquitoes resistant to permethrin, the level of expression of other *GSTe*s was higher in DDT-resistant mosquitoes. For Cameroon, *GSTe2*, *GSTe4* and *GSTe3* were more overexpressed in DDT-alive females. Overall, these results showed that the *GSTe*s are overexpressed mainly in DDT-resistant mosquitoes compared to permethrin and unexposed mosquitoes.

### 3.3. Functional Validation of the Role of GSTe Genes in Insecticide Resistance

#### 3.3.1. Confirmation of *GSTe* Knockdown Effect by qRT-PCR

**Figure 3:**
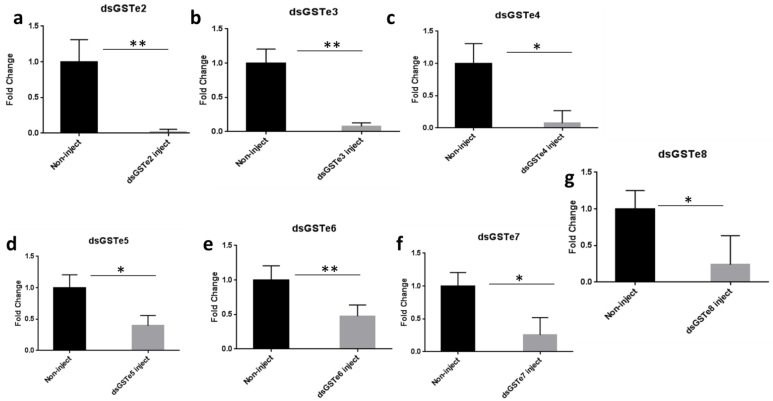
Confirmation of *GSTe* knockdown effect by quantitative RT-PCR between non-exposed double-strand injected and non-injected mosquitoes of the same age (**a**) ds*GSTe*2, (**b**) ds*GSTe*3, (**c**) ds*GSTe*4, (**d**) ds*GSTe*5, (**e**) ds*GSTe*6, (**f**) ds*GSTe*7, (**g**) ds*GSTe*8,. There is low expression of all *GSTe* injected mosquitoes compared to the non-injected mosquitoes. dsRNA injection significantly reduces the expression of the GST epsilon genes. * = *p* < 0.05, and ** = *p* < 0.01.

To confirm whether the injection of ds*GSTe*s did knock-down the expression of *GSTe* genes in vivo in mosquitoes, qPCR was performed using the cDNA from unexposed ds*GSTe*s (injected) and non-injected mosquitoes with the primers of each *GSTe*s cluster, using actin5C and RPS7 as housekeeping genes. As shown in Figure 3, we noticed a significant partial reduction in *GSTe*s gene expression when comparing control non-exposed and double-strand *GSTe*-injected mosquitoes 4 days post-injection *p* = 0.0024 (*GSTe2*), 0.0014 (*GSTe3*), 0.0377 (*GSTe4*), 0.0422 (*GSTe5*), 0.0014 (*GSTe6*), 0.0387 (*GSTe7*) and 0.0241 (*GSTe8*). This low expression of all the *GSTe*s in mosquitoes injected compared to the non-injected supports that in vivo injection of dsRNA significantly reduces the expression of GST epsilon genes in the Mibellon *An. funestus* mosquitoes.

#### 3.3.2. *GSTe* Knockdown Increases Susceptibility to Permethrin

**Figure 4:**
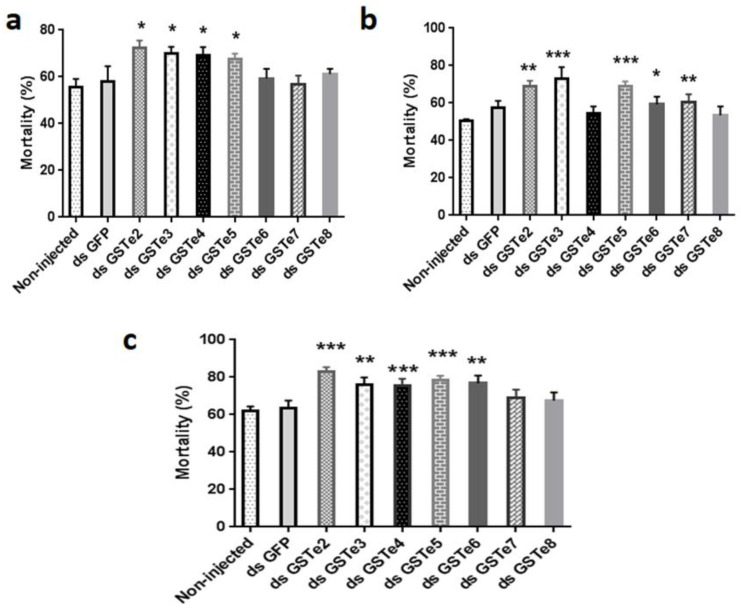
GSTe knockdown increases susceptibility to insecticides; bioassay result of mosquitoes after injection and exposure to (**a**) permethrin, (**b**) deltametrin and (**c**) DDT. * = *p* < 0.05, ** = *p* < 0.01, and *** = *p* < 0.001.

Significantly higher mortalities were observed in ds*GSTe*2-injected mosquitoes (Mibellon *An. funestus*) exposed to permethrin (72.46% ± 3.09; *p* = 0.0052), compared with non-injected control mosquitoes, with mortalities of 55.50% ± 3.59 (Figure 4a). Similar patterns were seen with *GSTe3* (mortality in dsGSTe3 = 70.09 ± 2.75%; *p* = 0.009), *GSTe4* (69.01 ± 3.70%; *p* = 0.017) and *GSTe5* (67.61 ± 4.41%; *p* = 0.018). Mortality with control mosquitoes injected with dsGFP is also 58.09% ± 6.41. No significant difference was obtained between the mortality of mosquitoes injected with ds*GSTe*6 (59.28 ± 4.14%; *p* = 0.25), ds*GSTe*7 (56.93 ± 3.63%; *p* = 0.39) and ds*GSTe*8 (61.25 ± 2.16; *p* = 0.13) compared to control mosquitoes exposed to permethrin. The mortality rate did not vary significantly between non-injected and mosquitoes injected with dsGFP, indicating that injection did not affect the survival of mosquitoes. These findings showed that GSTe2 3, 4 and 5 are playing a major role in permethrin resistance in this *An. funestus* population.

#### 3.3.3. *GSTe* Knockdown Increases Susceptibility to Deltamethrin

Exposure of ds*GSTe*s-injected mosquitoes to deltamethrin (Figure 4b) revealed that the mortality rate was higher in ds*GSTe*2 (69.06 ± 2.92%; *p* = 0.026), ds*GSTe*3 (72.87 ± 6.29%; *p* = 0.0001), ds*GSTe*5 (68.92 ± 2.58%; *p* = 0.000038), ds*GSTe*6 (59.28 ± 4.14%; *p* = 0.018) and ds*GSTe*7-injected mosquitoes (60.53 ± 1.63%; *p* = 0.00039) compared to non-injected (50.25% ± 1.18) and dsGFP-injected mosquitoes (57.40 ± 3.78%). No significant differences were observed in *GSTe*4 (54.41 ± 3.68%; *p* = 0.11) and *GSTe*8-injected mosquitoes (53.49 ± 4.73%; *p* = 0.22) compared to the control non-injected ones. This result showed that knock-down of *GSTe*s increased mosquito susceptibility to deltamethrin except for *GSTe4* and *GSTe8* where no impact was observed.

#### 3.3.4. *GSTe* Knockdown Increases Susceptibility to DDT

By exposing double-strand *GSTe*s-injected mosquitoes to DDT, we observed a significant difference between the mortality rates of *GSTe2* (83.05 ± 2.39%; *p* = 0.0001), *GSTe3* (75.87 ± 3.99%; *p* = 0.0036), *GSTe4* (75.45 ± 3.72%; *p* = 0.00052), *GSTe5* (78.68 ± 2.11%; *p* = 0.00038) and *GSTe6* (76.78 ± 4.19%; *p* = 0.003) knocked-down mosquitoes compared to the non-injected mosquitoes (62.21 ± 2.16%) and dsGFP-injected mosquitoes (63.52 ± 4.01%) (Figure 4c). Knockdowns of *GSTe*s genes family in Mibellon mosquitoes increased resistance to DDT excepted for *GSTe7* and *GSTe8*. From these results, we concluded that GST epsilon genes are playing a role in *An. funestus* insecticide resistance.

## 4. Discussion

Understanding of the dynamics of resistance development, the potential of some candidate genes to confer cross-resistance between insecticide classes and designing suitable diagnostic tools are crucial for malaria control. The major genes linked to metabolic insecticide resistance in the major malaria vector *An. funestus*, include CYP450s, GSTs and carboxylesterase. The characterisation of these major genes provides important information enabling the understanding and the dynamic of resistance development and how and where it spreads, facilitating its management.

In this study, comparative RNAseq-based transcriptomic profiling across Africa showed key differences in the level of expression of GSTs across Africa, including the epsilon, delta and the theta class. These genes are highly expressed in West and Central Africa, in contrast to southern Africa where GSTs are found to be less overexpressed, contrary to P450s, especially the duplicated *CYP6P9a* and *-b* genes, which are highly overexpressed in this region [32]. The greater over-expression of P450s genes observed when comparing transcriptomic profiling of DDT resistance across Africa do not necessarily indicate that this enzyme family plays the major role in DDT resistance but could be a result of the multiple resistance observed in these mosquito population with pyrethroids and carbamate resistance as reported. This is supported by the fact that previous studies, such as in *An. gambiae,* have showed little difference in mortality between bioassays done with DDT alone or after pre-exposure to the synergist PBO [36], showing that P450s may not be the main drivers of DDT resistance but more likely, GSTs in the absence of kdr as seen in *An. funestus* [37]. The difference in gene expression pattern observed between *An. funestus* populations is in line with previous studies done in *An. funestus* [15,32,38,39]. Transcriptional profiling using microarrays/qPCR has established higher overexpression of GSTs in populations from West Africa (Benin) compared with populations from Uganda and Malawi [15]. Among GSTs, the epsilon and delta families are the most expressed as seen also in other mosquito species [13,40], but the GST epsilon cluster is more consistently over-expressed across Africa as previously reported in *An. funestus* permethrin resistance [32]. All genes of the GST epsilon cluster are observed to be overexpressed in Africa in resistant population of *An. funestus*, but at different levels, except for *GSTe8.* This association between overexpression of *GSTe*s and DDT resistance could be explained by the fact that GST plays a role in oxidative stress and its expression is elevated in the response of oxidative damage caused by xenobiotic [41]. Besides cytochrome P450 and GSTs, other gene families were involved in DDT resistance including carboxylesterases, sulfotransferase, ATP-binding factor, UDP-glucuronosyltransferase, and metalloproteinase, and the same pattern were observed in *An. gambiae* using microarray [42,43].

Silencing of *An. funestus GSTe* genes supported the role that these genes played in DDT, as well as cross-resistance they confer to pyrethroids. This is in agreement with the findings of Riveron and colleagues [16] who also revealed a cross-resistance between these insecticide classes for *GSTe2* using GAL4/UAS transgenic expression in Drosophila and also the association studies of the *L119F-GSTe2* genotypes and resistance phenotypes [14,16]. While the work of Riveron and other researchers [18] characterized *GSTe2,* in particular, in this study, other epsilon GSTs were investigated, confirming their role in DDT and pyrethroid resistance. This cross-resistance could be either by directly metabolising the insecticides or conferring protection from oxidative stress induced by pyrethroids using a mechanism of sequestration [44]. It could also act by detoxifying/scavenging the secondary product generated by reactive oxygen species or by directly metabolizing 4-hydroxy-nonenal, an end product of lipid peroxidation, through conjugation [45].

This study has shown that knockdown of *GSTe*s in *An. funestus* significantly increase susceptibility to type I and II pyrethroids, suggesting that the overexpression of *GSTe*s could confer permethrin and deltamethrin resistance. This observation agrees with the results of Lumjuan in 2011, where they proved that partial knockdown of *GSTe2* and *GSTe7* in *Aedes aegypti* significantly increased susceptibility to DDT and deltamethrin [46]. This study has also supported that gene silencing through RNAi technique is a good tool to validate the role of candidate genes in insecticide resistance notably for *GSTe*s.

## 5. Conclusions

The results have identified genes associated with DDT resistance in the major malaria vector *An. funestus* Africa-wide. The gene families identified as overexpressed include the cytochrome P450s commonly known to confer resistance to wide range of public health insecticide in malaria vector, carboxylesterase and glutathione-S-transferase, which are the major focus of this study. This has established that *GSTe* genes are differentially over-expressed in resistant populations of *An. funestus* across Africa, with the genes consistently more overexpressed in Western and Central Africa compared to East and Southern Africa, consistent with the higher DDT resistance known in the *An. funestus* populations from West Africa. In addition, the *GSTe2*, *GSTe3*, *GSTe4*, *GSTe5* and *GSTe6* genes were shown to confer cross-resistance to permethrin, deltamethrin and DDT explaining the multiple resistance observed in the field, highlighting the complexity of resistance and challenges associated with malaria vector control. Further studies need to be performed to detect the genetic variants associated with the resistance conferred by the GST epsilon genes as done previously for *GSTe2*.

## Figures and Tables

**Table 1:** Detoxification genes differentially expressed in Cameroon *An. funestus* between different comparisons at false discovery rate (FDR) < 0.05 and fold change (FC) > 1.5 for genes induced upon exposure and constitutive expression (R-C) or FC > 2 for constitutive differential expression (C-S) and genes induced upon exposure and constitutive expression (R-S).

Gene ID	R-C	C-S	R-S	Description
AFUN002514	2.8934693		3.4858232	Carboxylesterase, COEunkn
AFUN019220		3.755253	4.1573305	ABC transporter family A
AFUN015966		15.648611	19.80706	Cytochrome P450, *CYP325A*
AFUN015889		2.4889274	3.1110048	Cytochrome P450, *CYP6P9b*
AFUN015888		5.4777937	8.212809	Cytochrome P450, *CYP6P5*
AFUN005715		2.3714201	2.252707	Cytochrome P450, *CYP315A1*
AFUN011266		2.67154	2.5182736	UDP-glucuronosyltransferase 3A1
AFUN022201		10.439024	9.539281	Glutathione peroxidase, Short
AFUN015807		2.3210542	2.5459306	Glutathione S-transferase, *GSTE1*
AFUN015808		2.4095497	2.4903498	Glutathione S-transferase, *GSTE3*
AFUN015839		2.004527	2.098784	Glutathione S-transferase, *GSTD3*
AFUN016008		3.0603988	3.182327	Glutathione S-transferase, *GSTE6*
AFUN007291		2.4175029	2.2559748	Glutathione S-transferase, *GSTT2*
AFUN015809		3.4979115	4.4708834	Glutathione S-transferase epsilon 2, *GSTE2*
AFUN016207		2.297438	2.2629843	Sulfotransferase family cytosolic 1B member 1; Short
AFUN008941		2.1940587		ABC transporter family C
AFUN016311		2.155015		Carboxylesterase, *COEBE3C*
AFUN002978		2.058842		Cytochrome P450, *CYP314A1*
AFUN001383	1.6504059			Cytochrome P450, *CYP9J5*
AFUN002602			2.0370903	Cytochrome *b*-561 domain containing protein
AFUN006858			2.3245227	Cytochrome P450, *CYP306A1*

**Table 2:** Detoxification genes differentially expressed in Ghana between different comparisons at FDR < 0.05 and FC > 1.5 for R-C or FC > 2 for C-S and R-S.

Genes ID	R-C	C-S	R-S	Description
AFUN015830	1.5176085	2.6203227	3.976624	Cytochrome P450, *CYP325C*
AFUN015894	1.7204243	4.830863	8.311134	Cytochrome P450, *CYP4H26*
AFUN019348	2.2147367	5.578066	12.353948	Cytochrome P450, *CYP325B*
AFUN015767	1.5001847	2.0304153	3.0459979	Glutathione S-transferase, *GSTD11*
AFUN019220		3.5253153	3.8225787	ABC transporter family A
AFUN002796		2.9251132	2.0283942	Sub-family G member 1
AFUN016367		2.291519	2.4250317	Carboxylesterase, *COEJHE4E*
AFUN019401		2.4856389	2.5770857	Cytochrome P450, *CYP6M4*
AFUN004316		4.7323303	7.0621023	Cytochrome P450, *CYP4H17*
AFUN006135		2.4041867	2.1305745	Cytochrome P450, *CYP4C36*
AFUN007549		2.8564737	2.3530338	Cytochrome P450, *CYP9K1*
AFUN020895		41.131523	30.434338	Cytochrome P450, *CYP6P4*
AFUN019365		17.181461	13.95981	Cytochrome P450, *CYP6P4*
AFUN001383		3.2154377	3.093064	Cytochrome P450, CYP9J5
AFUN015792		3.5820096	5.169663	Cytochrome P450, CYP6P9A
AFUN015889		6.209893	6.1071906	Cytochrome P450, *CYP6P9b*
AFUN015888		7.485999	6.140407	Cytochrome P450, *CYP6P5*
AFUN006858		2.186648	2.5465918	Cytochrome P450, *CYP306A1*
AFUN015795		2.3312643	2.0709553	Cytochrome P450, *CYP6M3*
AFUN005715		2.0942264	2.0190682	Cytochrome P450, *CYP315A1*
AFUN019567		6.2830515	6.3453665	Cytochrome P450, *CYP4H18*
AFUN016456		2.2823555	2.137907	D7 short form salivary protein
AFUN011266		2.815869	3.8286955	UDP-glucuronosyltransferase 3A1
AFUN015807		3.92929	4.207333	Glutathione S-transferase, *GSTE1*
AFUN015808		2.9914625	2.6008918	Glutathione S-transferase, *GSTE3*
AFUN015810		3.4769716	2.789998	Glutathione S-transferase, *GSTE4*
AFUN015811		2.9477298	2.0025258	Glutathione S-transferase, *GSTE5*
AFUN015839		4.0754375	3.4843476	Glutathione S-transferase, *GSTD3*
AFUN015840		3.6762526	4.7960463	Glutathione S-transferase, *GSTD10*
AFUN016008		3.3691344	3.113351	Glutathione S-transferase, *GSTE6*
AFUN001774		2.3985367	2.0373375	Glutathione s-transferase, *GSTE7*
AFUN015809		9.269093	7.5253835	Glutathione S-transferase, *GSTE2*
AFUN008239		2.6680195	2.1065671	Sulfotransferase
AFUN016207		2.6827705	2.3955698	Sulfotransferase
AFUN010696	2.5507598		3.926304	Cytochrome b5 domain-containing protein 1
AFUN015963	1.5521032		2.153224	Cytochrome P450, *CYP6R1*
AFUN016458	2.4133177		3.0593035	D7 short form salivary protein
AFUN008338	2.4747007		3.1143987	Sulfotransferase 1C4
AFUN008852		2.1302986		Glycosyltransferase
AFUN008941	2.0767095			ABC transporter family C
AFUN021098	2.351223			Cytochrome P450, *CYP4H19*
AFUN015909	2.0092266			Cytochrome P450, *CYP305A3*
AFUN015776	2.0201716			Cytochrome P450, *CYP12F1*
AFUN001382	2.1402965			Cytochrome P450, *CYP9J5*
AFUN019845	2.144599			Glucosyl/glucuronosyl transferases
AFUN016010	2.0161536			Glutathione S-transferase, *GSTD1*
AFUN008819	2.0289357			Glutathione transferase microsomal, *GSTMS3*
AFUN008941			2.0767095	ABC transporter family C
AFUN021098			2.351223	Cytochrome P450, *CYP4H19*
AFUN015909			2.0092266	Cytochrome P450, *CYP305A3*
AFUN015776			2.0201716	Cytochrome P450, *CYP12F1*
AFUN001382			2.1402965	Cytochrome P450, *CYP9J5*
AFUN019845			2.144599	UDP-glucuronosyltransferase 2C1
AFUN016010			2.0161536	Glutathione-S-transferase, *GSTD1*
AFUN008819			2.0289357	Glutathione-S-transferase microsomal, *GSTMS3*

**Table 3:** Detoxification genes differentially expressed in Malawi between different comparisons at FDR < 0.05 and FC > 1.5 for R-C or FC > 2 for C-S and R-S.

Genes ID	R-C	C-S	R-S	Description
AFUN019523	1.507577	4.439413	6.692756	Cytochrome P450, *CYP325J1*
AFUN015889	2.5220747	16.651278	41.99576	Cytochrome P450, *CYP6P9b*
AFUN019220		3.1381407	2.659554	ABC transporter family A
AFUN016265		3.7209604	4.703569	Carboxylic ester hydrolase
AFUN019401		2.8624895	2.7167373	Cytochrome P450, *CYP6M4*
AFUN015801		2.299937	2.2996929	Cytochrome P450, *CYP6P2*
AFUN020895		6.031834	6.101497	Cytochrome P450, *CYP6P4*
AFUN015792		53.420876	49.825886	Cytochrome P450, *CYP6P9a*
AFUN002978		2.048785	2.5891387	Cytochrome P450, *CYP314A1*
AFUN010918		2.2058907	2.3527682	Cytochrome P450, *CYP6N1*
AFUN011266		2.2034643	3.0369558	UDP-glucuronosyltransferase 3A1
AFUN015839		5.0719476	3.63867	Glutathione S-transferase, *GSTD3*
AFUN016008		2.8427892	3.0745156	Glutathione S-transferase, *GSTE6*
AFUN016207		2.7247274	2.1642609	Sulfotransferase
AFUN015907		2.2404246		Cytochrome P450, *CYP305A3*
AFUN015785		2.1118221		Cytochrome P450, *CYP6AA2*
AFUN015807		2.5705426		Glutathione S-transferase, *GSTE1*
AFUN004322			2.2281806	Cytochrome b5
AFUN006135			2.3192496	Cytochrome P450, *CYP4C36*
AFUN007549			2.2119997	Cytochrome P450, *CYP9K1*
AFUN016010			2.1173494	Glutathione S-transferase, *GSTD1*
AFUN015767			2.0815623	Glutathione S-transferase, *GSTD11*
AFUN015811			2.2649431	Glutathione S-transferase, *GSTE5*
AFUN015809			2.1379018	Glutathione S-transferase epsilon 2, *GSTE2*
AFUN007291			2.0031114	Glutathione S-transferase, *GSTT2*
AFUN008239			2.3338351	Sulfotransferase
AFUN016367	1.8275834			Carboxylesterase, *COEJHE4E*
AFUN016209	1.5197136			Sulfotransferase

**Table 4:** Detoxification genes differentially expressed in Uganda between different comparisons at FDR < 0.05 and FC > 1.5 for R-C or FC > 2 for C-S and R-S.

Genes ID	R-C	C-S	R-S	Description
AFUN019220		2.9239964	2.9306972	ABC transporter family A
AFUN000421		2.0856595	2.4146874	Carboxylesterase, *COEBE4C*
AFUN015777		3.8722458	3.1892729	Cytochrome P450, *CYP4C26*
AFUN015888		3.0184207	3.1936443	Cytochrome P450, *CYP6P5*
AFUN015839		2.9561374	2.1264198	Glutathione S-transferase, *GSTD3*
AFUN016008		3.5037928	2.787403	Glutathione S-transferase, *GSTE6*
AFUN016207		2.6702209	2.6851285	Sulfotransferase
AFUN002796		2.711219		ABC transporter sub-family G member 1;
AFUN019365		2.3760457		Cytochrome P450, *CYP6P4*
AFUN006858		2.0747128		Cytochrome P450, *CYP306A1*
AFUN011518		2.2290916		Cytochrome c oxidase assembly factor 5
AFUN015735	2.302202			Cytochrome P450, *CYP49A1*
AFUN007537	1.6417327			ABC transporter *ABCF3*
AFUN019517	1.9148197			Cytochrome P450, *CYP325J1*
AFUN019708	1.8381125			Sulfotransferase 2
AFUN019950	1.8083609			Carboxylesterase, *COE2580*
AFUN020232	1.5087312			Metalloproteinase domain-containing protein
AFUN020202	1.7750088			NADPH oxidase 5
AFUN015907			2.350208	Cytochrome P450, *CYP305A3*
AFUN005715			2.1279395	Cytochrome P450, *CYP315A1*
AFUN016010			2.034498	Glutathione S-transferase, *GSTD1*

## Data Availability

Raw data from RNAseq is deposited on sequence archive, with the following link: https://www.ebi.ac.uk/ena/browser/view/PRJEB24351, accessed on 11 February 2021. All other data is present in the manuscript and supplemental files.

## Supplementary Materials

The following are available online at https://www.mdpi.com/article/10.3390/genes12040561/s1. Supplementary file 1: Genes differentially expressed when comparing DDT (dichlo-ro-diphenyl-trichloroethane) vs permethrin-resistant individuals using RNA-seq (RNA sequenc-ing). Table S1.1: Genes differentially expressed in Cameroon when comparing DDT vs Perm resistant mosquitoes, Table S1.2: Genes differentially expressed in Ghana when comparing DDT vs Perm resistant mosquitoes, Table S1.3: Genes differentially expressed in Uganda when comparing DDT vs Perm resistant mosquitoes, Table S1.4: Genes differentially expressed in Malawi when comparing DDT vs Perm resistant mosquitoes, Table S1.5: List of genes differentially over-expressed across Africa when comparing resistant population against Permethrin and DDT using RNAseq. Supplementary file 2: List of primers used. Table S2.1: list of primers used to evaluate GSTes expression profile across Africa, Table S2.2: list of primers used for double-stranded RNA synthesis.
