# Supplementary material for: Genome-Wide Transcriptional Analysis and Functional Validation Linked a Cluster of Epsilon Glutathione S-Transferases with Insecticide Resistance in the Major Malaria Vector Anopheles funestus across Africa
Source: Genes (Basel). 2021 Apr 13;12(4):561. doi: 10.3390/genes12040561 (PMC8069850; doi:10.3390/genes12040561)
Supplement: Supplementary file 1 [file genes-12-00561-s001.zip › genes-1127066-supplementary/Kouamo et al manuscript Supplementary file 1.docx]

**Supplementary File 1: Genes differentially expressed when comparing DDT vs Perm resistant individuals using RNAseq**

**Supplementary Table S1.1 :Genes differentially expressed in Cameroon when comparing DDT vs Perm resistant mosquitoes**

**FC ([Rperm]: Fold Change in Permethrin Resistant mosquitoes**

**FC[Rddt]): Fold hange in DDT resistant mosquitoes**

|  | **Gene ID** | **FC ([Rp] vs [Rddt])** | **Description** |
| --- | --- | --- | --- |
| **Upregulated** | AFUN017741 | 15.587959 | Eukaryotic large subunit ribosomal RNA |
|  | AFUN017765 | 24.807547 | Eukaryotic large subunit ribosomal RNA |
|  | AFUN017743 | 16.25705 | Eukaryotic large subunit ribosomal RNA |
|  | AFUN017737 | 14.020776 | Eukaryotic large subunit ribosomal RNA |
|  | AFUN017574 | 7.613681 | Eukaryotic small subunit ribosomal RNA |
|  | AFUN017756 | 5.596585 | Eukaryotic large subunit ribosomal RNA |
|  | AFUN017336 | 11.796383 | Eukaryotic small subunit ribosomal RNA |
|  | AFUN010220 | 2.0599177 | Ornithine decarboxylase 1 |
|  | AFUN016252 | 2.2633421 | NA |
|  | AFUN021932 | 1.5017865 | heme peroxidase 14 |
|  | AFUN015966 | 1.5626017 | Cytochrome P450, *CYP325A* |
|  | AFUN014318 | 1.5864193 | NA |
|  | AFUN022209 | 1.7754643 | secretory phospholipase A2 |
|  | AFUN016787 | 1.8100652 | tRNA-Ser for anticodon AGA |
|  | AFUN016457 | 1.6353623 | D7 short form salivary protein |
|  | AFUN019636 | 2.390741 | NA |
|  | AFUN019590 | 2.1915233 | NA |
| **Downregulated** | AFUN018669 | -2.8696635 | NA |
|  | AFUN018844 | -1.6577433 | Coagulation factor, FA9 |
|  | AFUN002277 | -2.6796584 | odorant-binding protein |
|  | AFUN017623 | -2.2219305 | Eukaryotic small subunit ribosomal RNA |
|  | AFUN015275 | -9.692202 | NA |
|  | AFUN021561 | -2.0741463 | Probable prefoldin subunit, PFD6 |
|  | AFUN018859 | -2.5157685 | Hexamerin-1.1 |
|  | AFUN017108 | -3.2575352 | tRNA-Val for anticodon CAC |
|  | AFUN021871 | -3.1633754 | NA |
|  | AFUN019223 | -2.1574721 | BOVINRecName, TEKT4 |
|  | AFUN016158 | -1.5113468 | UDP-glucuronosyltransferase, *UD2A3* |
|  | AFUN016757 | -2.3624623 | tRNA-Thr for anticodon AGU |
|  | AFUN019317 | -2.00401 | Transmembrane emp24 domain-containing protein bai |

**Supplementary Table S1.2.:Genes differentially expressed in Ghana when comparing DDT vs Perm resistant mosquitoes**

|  | **Gene ID** | **FC ([Rp] vs [Rddt])** | **Description** |  |  | |
| --- | --- | --- | --- | --- | --- | --- |
| **Upregulated** | AFUN021325 | 1.5028327 | hemolymph protein, P27K |  |  |  |
|  | AFUN015186 | 1.6101412 | GSK3-beta interaction protein, GSKIP |  |  |  |
|  | AFUN019847 | 1.6285688 | Heat shock protein, HSP90 |  |  |  |
|  | AFUN017484 | 2.0182884 | Eukaryotic small subunit ribosomal RNA |  |  |  |
|  | AFUN018423 | 1.8434883 | tRNA-Ser for anticodon CGA |  |  |  |
|  | AFUN008339 | 1.6796623 | glucose-regulated protein, GRP-94 |  |  |  |
|  | AFUN019660 | 1.5367527 | Ubiquitin-related, UBIQ1 |  | |  |
|  | AFUN011065 | 1.6808943 | NA |  |  | |
|  | AFUN000046 | 1.5007594 | protein disulfide-isomerase, PDIA6 |  |  |  |
|  | AFUN020453 | 1.6042627 | NA |  |  | |
|  | AFUN001129 | 1.9502834 | Ecdysteroid-regulated protein |  |  |  |
|  | AFUN015292 | 1.5647336 | Ice nucleation protein |  | |  |
|  | AFUN003291 | 1.5232863 | NA |  |  | |
|  | AFUN019689 | 1.7178811 | Ribonuclease, RNH1 |  | |  |
|  | AFUN016757 | 1.5662625 | tRNA-Thr for anticodon AGU |  |  |  |
|  | AFUN019720 | 1.5636328 | Transcription initiation factor, *TAF13* |  |  |  |
|  | AFUN014924 | 1.531763 | NA |  |  | |
| **Downregulated** | AFUN021134 | -2.0330873 | cellular retinaldehyde binding protein |  |  |  |
|  | AFUN009738 | -2.4548316 | odorant-binding protein, *OBP47* |  |  |  |
|  | AFUN019089 | -2.2794962 | NA |  |  | |
|  | AFUN018991 | -5.7070584 | NA |  |  | |
|  | AFUN010097 | -2.0184186 | NA |  |  | |
|  | AFUN017021 | -2.6483355 | mir-9/mir-79 microRNA precursor family |  |  |  |
|  | AFUN018399 | -2.2500498 | tRNA-His for anticodon GUG |  |  |  |
|  | AFUN019021 | -2.086617 | Collagenase precursor |  | |  |
|  | AFUN008749 | -3.1838536 | NA |  |  | |
|  | AFUN018858 | -2.1941888 | NA |  |  | |
|  | AFUN003966 | -2.339823 | Class B Scavenger Receptor |  |  |  |
|  | AFUN009897 | -2.704369 | NA |  |  | |
|  | AFUN011132 | -2.2574177 | Pickpocket protein, PPK28 |  |  |  |
|  | AFUN019167 | -13.814865 | NA |  |  | |
|  | AFUN005380 | -2.43218 | NA |  |  | |
|  | AFUN021561 | -2.5240476 | Probable prefoldin subunit 6 |  |  |  |
|  | AFUN021699 | -2.6736994 | NA |  |  | |
|  | AFUN007532 | -2.68601 | Angiopoietin-related protein 2 |  |  |  |
|  | AFUN010204 | -4.7623925 | NA |  |  | |
|  | AFUN019628 | -5.293229 | NA |  |  | |
|  | AFUN015929 | -2.07252 | NA |  |  | |
|  | AFUN003002 | -2.0492675 | Class B Scavenger Receptor (CD36 domain), *SCRB1* |  |  |  |
|  | AFUN022201 | -1.6014247 | glutathione peroxidase 3 |  |  |  |
|  | AFUN015198 | -2.045808 | NA |  |  | |
|  | AFUN016525 | -4.158567 | NA |  |  | |
|  | AFUN019186 | -2.121283 | NA |  |  | |
|  | AFUN021616 | -2.2311869 | General odorant-binding protein, *OBP72* |  |  |  |
|  | AFUN022042 | -2.4795148 | Pickpocket protein, *PPK11* |  |  |  |
|  | AFUN019411 | -7.0906916 | NA |  |  | |
|  | AFUN016737 | -2.9234557 | tRNA-His for anticodon GUG |  |  |  |
|  | AFUN021369 | -7.0147233 | metalloproteinase, *NAS1* |  | |  |
|  | AFUN008922 | -3.8918836 | NA |  |  | |
|  | AFUN006196 | -3.8422039 | Mammalian branch point-binding protein, *SF01* |  |  |  |
|  | AFUN019636 | -2.1408179 | NA |  |  | |
|  | AFUN003809 | -2.0026965 | Putative odorant-binding protein, OB10 |  |  |  |
|  | AFUN014165 | -4.7097044 | NA |  |  | |
|  | AFUN006855 | -3.5193913 | NA |  |  | |
|  | AFUN021098 | -1.5484301 | cytochrome P450, *CYP4H19* |  |  |  |

**Supplementary Table S1.3 : Genes differentially expressed in Uganda when comparing DDT vs Perm resistant mosquitoes**

|  | **Gene ID** | **FC ([Rperm] vs [Rddt])** | **Description** |
| --- | --- | --- | --- |
| **Upregulated** | AFUN021811 | 1.6029985 | Farnesol dehydrogenase, *SDR1* |
|  | AFUN018396 | 2.8209891 | tRNA-Arg for anticodon UCG |
|  | AFUN014447 | 2.6519194 | Spondin Precursor |
|  | AFUN019038 | 2.9406083 | NA |
|  | AFUN022115 | 2.9183872 | NA |
|  | AFUN017755 | 2.116733 | Eukaryotic large subunit ribosomal RNA |
|  | AFUN017649 | 3.2072444 | Eukaryotic large subunit ribosomal RNA |
|  | AFUN017328 | 47.045692 | Eukaryotic small subunit ribosomal RNA |
|  | AFUN017404 | 2.2988944 | Eukaryotic large subunit ribosomal RNA |
|  | AFUN019007 | 2.5040815 | NA |
|  | AFUN001292 | 2.464326 | NA |
|  | AFUN016153 | 2.8789062 | Putative serine protease, *K12H4* |
|  | AFUN016265 | 1.7766759 | Carboxylic ester hydrolase |
|  | AFUN019636 | 2.897606 | NA |
|  | AFUN003809 | 1.7066069 | chemosensory protein, *CSP3* |
| **Downregulated** | AFUN018455 | -2.716616 | tRNA-Tyr for anticodon GUA |
|  | AFUN008656 | -2.74286 | alpha-crystallin B chain |
|  | AFUN021807 | -2.2841327 | Farnesol dehydrogenase, SDR1 |
|  | AFUN017103 | -11.194956 | 5.8S ribosomal RNA |
|  | AFUN019152 | -2.3926342 | Chymotrypsin |
|  | AFUN010685 | -2.6143346 | cuticular protein RR-1 family, *CUD2* |
|  | AFUN018546 | -2.1816304 | 39S ribosomal protein L42 |
|  | AFUN021434 | -4.2670565 | Chymotrypsin-elastase inhibitor ixodidin |
|  | AFUN000679 | -1.7563882 | UDP-glucuronosyltransferase, *UD18* |
|  | AFUN017551 | -20.365372 | Regulator of rDNA transcription protein, *RRT15* |
|  | AFUN017609 | -5.4200435 | Eukaryotic large subunit ribosomal RNA |
|  | AFUN017348 | -71.82415 | Eukaryotic small subunit ribosomal RNA |
|  | AFUN020398 | -2.5339777 | Potassium voltage-gated channel subfamily, *KCNQ1* |
|  | AFUN002510 | -2.1183908 | phospholipase b, *plb1* |
|  | AFUN019137 | -1.5197557 | ATP-binding cassette sub-family A member 3 |
|  | AFUN019218 | -1.7663617 | Cytochrome P450, *CYP325J1* |
|  | AFUN005103 | -1.5033096 | Cytochrome P450, *CYP325F1* |
|  | AFUN009877 | -1.9640274 | cytochrome P450, *CYP6Z4* |
|  | AFUN007893 | -1.5197845 | Argininosuccinate synthase |
|  | AFUN003690 | -1.6836315 | Cytochrome P450, *CYP4H14* |
|  | AFUN015292 | -14.83721 | Ice nucleation protein |
|  | AFUN019513 | -4.584 | Heat shock protein 70 B2 |
|  | AFUN021369 | -2.9131968 | Zinc metalloproteinase nas-1 |
|  | AFUN019428 | -2.4221325 | Adult cuticle protein 1 |
|  | AFUN021584 | -2.2233737 | Serine proteinase stubble |
|  | AFUN020965 | -3.2765036 | Lactosylceramide 4-alpha-galactosyltransferase |
|  |  |  |  |

**Supplementary Table S1.4 : Genes differentially expressed in Malawi when comparing DDT vs Perm resistant mosquitoes**

|  | **Gene ID** | **FC ([RDDT] vs [RPerm])** | **Description** |
| --- | --- | --- | --- |
| **Upregulated** | AFUN018876 | 2.1157522 | akirin |
|  | AFUN022342 | 2.2446945 | purine-nucleoside phosphorylase |
|  | AFUN011532 | 2.4156828 | NA |
|  | AFUN000597 | 2.3362155 | Leucine-rich repeat transmembrane neuronal protein 2 |
|  | AFUN001928 | 3.1728892 | NA |
|  | AFUN010835 | 2.7511446 | odorant-binding protein |
|  | AFUN017595 | 2.3733828 | Eukaryotic large subunit ribosomal RNA |
|  | AFUN018819 | 2.3996756 | Protein PET100 homolog |
|  | AFUN022161 | 2.211473 | Transcription factor Adf-1 |
|  | AFUN022250 | 5.9530497 | Serine proteinase stubble |
|  | AFUN021561 | 3.4193678 | Probable prefoldin subunit 6 |
|  | AFUN002505 | 2.0876255 | Actin, cytoplasmic |
|  | AFUN017045 | 2.1718502 | U11 spliceosomal RNA |
|  | AFUN015889 | 1.9964486 | cytochrome P450, *CYP6P9b* |
|  | AFUN021056 | 1.6453323 | ATP-binding cassette sub-family A member 3 |
|  | AFUN018393 | 2.298294 | U1 spliceosomal RNA |
|  | AFUN008615 | 3.0601845 | Odorant-binding protein 1 |
|  | AFUN022201 | 1.8017676 | glutathione peroxidase |
|  | AFUN019513 | 7.402636 | Heat shock protein 70 B2 |
|  | AFUN019775 | 2.3728209 | Heat shock protein 70 A1 |
|  | AFUN014691 | 3.463066 | NA |
|  | AFUN021493 | 2.2095127 | phosphoribosylglycinamide formyltransfer |
|  | AFUN003809 | 2.3608007 | chemosensory protein 3, *CSP3* |
|  | AFUN003303 | 1.6750257 | Glutathione S-transferase, *GSTU3* |
| **Downregulated** | AFUN018981 | -2.4563937 | Transmembrane protease serine 11D |
|  | AFUN019165 | -2.153628 | E3 ubiquitin-protein ligase FANCL |
|  | AFUN007080 | -2.7267354 | short caspase, *CASPS6* |
|  | AFUN016052 | -3.2167156 | Carboxylic ester hydrolase, *COEBE2C* |
|  | AFUN017638 | -7.4041314 | Eukaryotic small subunit ribosomal RNA |
|  | AFUN017649 | -9.002373 | Eukaryotic large subunit ribosomal RNA |
|  | AFUN010296 | -3.172646 | Venom serine protease 34 |
|  | AFUN017092 | -6.0629582 | 5S ribosomal RNA |
|  | AFUN018655 | -4.517771 | odorant-binding protein |
|  | AFUN021904 | -3.1884897 | Niemann-Pick Type C-2 |
|  | AFUN015856 | -2.3378432 | Gustatory receptor, *Gr36* |
|  | AFUN021228 | -4.417828 | Golgi SNAP receptor complex member |
|  | AFUN008426 | -1.6448141 | glutathione S-transferase, *GSTU2* |
|  | AFUN015895 | -1.7465178 | Cytochrome P450, *CYP4H25* |
|  | AFUN015768 | -1.8057065 | glutathione S-transferase, *GSTD11* |
|  | AFUN020922 | -2.7718313 | cuticular protein RR-2 family, *CPR131* |
|  | AFUN016306 | -5.0771074 | Tubulin beta chain |
|  | AFUN004331 | -6.180799 | TBC1 domain family member |
|  | AFUN019401 | -1.6576518 | cytochrome P450, *CYP6M4* |
|  | AFUN005747 | -4.2846746 | cuticular protein 4 from fifty-one aa family, *CPF4* |

**Supplementary Table S1.5: List of genes differentially over-expressed across Africa when comparing resistant population against Permethrin and DDT using RNAseq**

| **Contry** | **Gene ID** | **Rperm] vs Rddt** | **Description** |
| --- | --- | --- | --- |
| **Cameroon** | AFUN015966 | 1.5626017 | Cytochrome P450, *CYP325A* |
|  | AFUN016457 | 1.6353623 | D7 short form salivary protein, D7r1 |
| **Uganda** | AFUN021811 | 1.6029985 | dehydrogenase/reductase SDR family member |
|  | AFUN016265 | 1.7766759 | Carboxylic ester hydrolase |
| **Ghana** | No |  |  |
| **Malawi** | AFUN021056 | 1.6453323 | ATP-binding cassette sub-family A member 3 |
|  | AFUN022201 | 1.8017676 | glutathione peroxidase, *GPXH3* |
|  | AFUN003303 | 1.6750257 | Glutathione S-transferase, *GSTU3* |
