# Supplementary material for: Genome-Wide Transcriptional Analysis and Functional Validation Linked a Cluster of Epsilon Glutathione S-Transferases with Insecticide Resistance in the Major Malaria Vector Anopheles funestus across Africa
Source: Genes (Basel). 2021 Apr 13;12(4):561. doi: 10.3390/genes12040561 (PMC8069850; doi:10.3390/genes12040561)
Supplement: Supplementary file 1 [file genes-12-00561-s001.zip › genes-1127066-supplementary/Kouamo et al manuscript Supplementary flies 2.docx]

**Supplementary file 2: List of primers uses**

**Supplementary able S2.1: list of primers used to evaluate GSTes expression profile across Africa**

| **Genes** | **Primers names** | **Sequences** | **Amplicon size (bp)** |
| --- | --- | --- | --- |
| *GSTe1* | qGSTe1 Foward | GCGCTTCATTACGGAACTGG | 151 |
|  | qGSTe1 Reverse | AAAATCAGCGATAGTCATTTGC |  |
| *GSTe2* | qGSTe2 Foward | GTTTGAAGCAGTTGCCATACTACGAGG | 101 |
|  | qGSTe2 Reverse | TCAAGCTTTAGCATTTTCCTCCTTTTTGGC |  |
| *GSTe3* | qGSTe3 Foward | CCAACCGTTTTGGCATATCT | 197 |
|  | qGSTe3 Reverse | TTTCTCACTGAGTACTTTGCCTTTT |  |
| *GSTe4* | qGSTe4 Foward | ATTGCGATTCTATGCGGAAC | 218 |
|  | qGSTe4 Reverse | GCTTCGGATAGTTGGCTTCA |  |
| *GSTe5* | qGSTe5 Foward | ACGAGCAGCTTAACCAGGAA | 187 |
|  | qGSTe5 Reverse | CCGTGTCTGCTTTTCTGTGA |  |
| *GSTe6* | qGSTe6 Foward | GGAGGTGACTGTTCGTGGAT | 161 |
|  | qGSTe6 Reverse | TCCGTCCTCGAAGGTAGGTA |  |
| *GSTe7* | qGSTe7 Foward | GGTACCGCTGGACGAGACTA | 182 |
|  | qGSTe7 Reverse | TTAGATTGGAGATTCTATCCCTTG |  |
| *GSTe8* | qGSTe8 Foward | TACCGATCGATGCCGAAC | 169 |
|  | qGSTe8 Reverse | TATTTTGCGCAACTTTTCCA |  |

**Supplementary Table S2.1: list of primers used for double-stranded RNA synthesis**

| **Genes** | **Primers names** | **Sequences** | **Amplicon size (bp)** |
| --- | --- | --- | --- |
| *GSTe2* | dsGSTe2 Foward | taatacgactcactatagggaga ACGGAACTTGTGTTTTTCGC | 473 |
|  | dsGSTe2 Reverse | taatacgactcactatagggaga TTGCTGGTGGGTGATAGTGA |  |
| *GSTe3* | dsGSTe3 Foward | taatacgactcactatagggaga CCGCAAGAGAAAATCGACTC | 308 |
|  | dsGSTe3 Reverse | taatacgactcactatagggaga TAACCTGGGACAAACCTTCG |  |
| *GSTe4* | dsGSTe4 Foward | taatacgactcactatagggaga ATTGCGATTCTATGCGGAAC | 413 |
|  | dsGSTe4 Reverse | taatacgactcactatagggaga CTTGCAGTTGATTTGCAGGA |  |
| *GSTe5* | dsGSTe5 Foward | taatacgactcactatagggaga TAGCTGCATCGCAACGATAG | 302 |
|  | dsGSTe5 Reverse | taatacgactcactatagggaga CCGTGTCTGCTTTTCTGTGA |  |
| *GSTe6* | dsGSTe6 Foward | taatacgactcactatagggaga GTGAAATCCCGGAGGAAAAT | 315 |
|  | dsGSTe6 Reverse | taatacgactcactatagggaga ACGCCTCAATCAAAGTGGTC |  |
| *GSTe7* | dsGSTe7 Foward | taatacgactcactatagggaga GAATCGTTATCACCGCCAGT | 359 |
|  | dsGSTe7 Reverse | taatacgactcactatagggaga CGGGAATTTAGTCTCGTCCA |  |
| *GSTe8* | dsGSTe8 Foward | taatacgactcactatagggaga AGCATACCTTTGCCATACCG | 364 |
|  | dsGSTe8 Reverse | taatacgactcactatagggaga CCAACTCGATTCTGGTCGTT |  |
